# Validation of the Arabic version of the “12-item short-form health survey” (SF-12) in a sample of Lebanese adults

**DOI:** 10.1186/s13690-021-00579-3

**Published:** 2021-04-23

**Authors:** Chadia Haddad, Hala Sacre, Sahar Obeid, Pascale Salameh, Souheil Hallit

**Affiliations:** 1 Research Department, Psychiatric Hospital of the Cross, P.O. Box 60096, Jal Eddib, Lebanon; 2 INSERM, Univ. Limoges, CH Esquirol, IRD, U1094 Tropical Neuroepidemiology, Institute of Epidemiology and Tropical Neurology, GEIST, Limoges, France; 3 INSPECT-LB (National Institute of Public Health, Clinical Epidemiology and Toxicology), Beirut, Lebanon; 4 Faculty of Arts and Sciences, Holy Spirit University of Kaslik (USEK), Jounieh, Lebanon; 5 Faculty of Pharmacy, Lebanese University, Beirut, Lebanon; 6 University of Nicosia Medical School, Nicosia, Cyprus; 7 Faculty of Medicine and Medical Sciences, Holy Spirit University of Kaslik (USEK), Jounieh, Lebanon

**Keywords:** Quality of life, SF-12, Validation, Arabic, Lebanon

## Abstract

**Background:**

In clinical practice, quality of life measures can be used alongside some types of assessment to give valuable information that can identify areas that influence an individual and help the clinician make the best healthcare choices. This study aimed to investigate the psychometric properties of the Arabic version of the 12-item short-form health survey (SF-12) in a sample of Lebanese adults.

**Methods:**

This cross-sectional study performed between July and November 2019 recruited 269 participants. Cronbach’s alpha was used to assess the reliability of the SF-12 questionnaire, and a factor analysis using the principal component analysis was performed to confirm its construct validity.

**Results:**

The mean score for the “physical component summary (PCS-12)” was 50.27 ± 8.94 (95 % CI: 49.18–51.36) and for the “Mental component summary (MCS-12)” was 44.95 ± 12.17 (95 % CI: 43.47–46.43). A satisfactory Cronbach’s alpha was found for the two components: MCS (α = 0.707) and PCS (α = 0.743). The principal component analysis converged over a two-factor solution (physical and mental), explaining a total variance of 55.75 %. Correlations between the SF-12 scales and single items were significant, showing a good construct validity. The “physical functioning”, “role physical”, “bodily pain”, and “general health” subscales were highly associated with “PCS-12”, while the “vitality”, “social functioning”, “role emotional”, and “mental health” subscales were more associated with MCS-12.

**Conclusions:**

The Arabic version of the SF-12 is a reliable, easy-to-use, and valid tool to measure health-related quality of life in the general population. Future studies using a larger sample size and focusing on questionnaire psychometric properties are necessary to confirm our findings.

## Background

According to the World Health Organization, quality of life (QOL) is “a multidimensional concept that typically contains domains related to physical, mental, emotional, and social functioning” [1]. It is the individual’s perception of their position in life within the context of culture and values in which they live and in relation to their needs, desires, standards, and concerns [1]. Measuring QOL in a population is essential to assess the burden of preventable diseases and injuries and provide valuable perceptions into the relations between QOL and risk factors [2]. It also helps identify subgroups with comparatively poor perceived well-being and allows direct measures to improve their outcomes and avoid more severe consequences [2]. The main reason for using QOL interventions is to ensure that care decisions and evaluations concentrate on the patient and not the illness [3]. In clinical practice, quality of life measures can be used alongside some types of assessment to give valuable information that can identify areas that influence an individual and help the clinician make the best healthcare choices [4]. These tools can serve to evaluate treatments owing to the details collected and thus may be used to measure QOL changes over the course of treatment [3, 4]. However, they cannot replace the assessment of disease-related outcomes but are an adjunct instead [4].

QOL is measured using two main approaches, generic and disease-specific, and many experts consider applying them concurrently [5]. The most commonly used generic instrument is the “Short-Form Health Survey (SF-36)”, a comprehensive, brief tool with high validity and reliability [6, 7]. A shorter alternate, the SF-12, was created to evaluate health-related QOL, which effectiveness and validity are well documented [8]. With its short administration time (less than two minutes), the SF-12 provides accurate and efficient information to assess physical and mental health QOL. It includes eight dimensions as the initial SF-36 instrument: general health perceptions (GH, 1 item), physical functioning (PF, 2 items), role limitations due to physical problems (RP, 2 items), bodily pain (BP, 1 item), vitality (VT, 1 item), social functioning (SF, 1 item), role limitations due to emotional problems (RE, 2 items), and mental health (MH, 2 items) [8]. The eight health concepts are represented by four 2-item dimensions (PF, RP, RE, and MH) and four single-item dimensions (BP, GH, VT, and SF) [8]. All twelve items can be summarized in two components, the “Physical Component Summary” (PCS-12) and “Mental Component Summary” (MCS-12) [8].

Several studies worldwide have explored SF-12 psychometric properties [9–15]. Overall, their findings indicate that the tool is accurate and reliable and can be used for QOL measurements in several population groups (general population and disease groups) [11, 16–19]. Contrary to the SF-36 that was translated into Arabic and validated in many countries such as Tunisia [20], Jordan [21], Saudi Arabia [22], and Lebanon [23], the SF-12 scale was only translated into Moroccan dialectical Arabic in Morocco [24], and classical Arabic in Saudi Arabia [25]. The Moroccan study included a sample of 141 subjects and revealed strong scale reliability and validity [24]. In Saudi Arabia, the study conducted among 432 participants tested the internal consistency of the translated questionnaire using the test-retest method and showed very good reliability with a Cronbach’s alpha value of 0.84 [25].

However, none of the studies that used the SF-12 have evaluated its psychometric properties in the Lebanese population [26, 27]. Thus, this study aimed to investigate the psychometric properties of the Arabic version of SF-12 in a sample of Lebanese adults.

## Methods

### Study design and sampling

A cross-sectional study performed between July and November 2019 recruited 269 community-dwelling participants. In 2019, the total population in Lebanon was 6,855,713 people, distributed across the 24 Districts (Caza) of the eight Lebanese Governorates (Mohafaza). A cluster sampling technique was used to choose a proportionate sample from the Lebanese villages (two per Caza), according to the list issued by the Central Agency of Statistics in Lebanon. Households were randomly chosen in the selected villages from a list generated by the municipalities. Data collection was done using face-to-face interviews with the participants who agreed to enroll. Eligibility was set at age 18 and above. People who had any cognitive impairment (difficulty filling up the survey) were excluded.

Prior to enrollment, the study objectives and methodology were explained to the participants, and their anonymity was guaranteed. Participation was voluntary, and participants received no incentives in return for joining the study.

### Sample size calculation

Based on a study by Comrey and Lee [28], 5–10 observations per item are needed for the scale validation process. Therefore, the sample size required to validate the SF-12 scale was 120 participants.

### Procedure

 Two study-independent personnel performed data collection through interviews with the participants. The survey was in Arabic (the native language in Lebanon) and consisted of closed-ended questions.

### Questionnaire

The questionnaire consisted of two sections. The first section collected information about the sociodemographic characteristics of the participants (age, gender, marital status, employment status, monthly income, and education level). Monthly income was categorized into low < 1,000 USD, intermediate 1,000–2,000 USD, and high > 2,000 USD. A self-reported binary question was used to evaluate the presence/absence of physical illness. A study-independent psychologist assessed the presence of mental illness.

The second section consisted of the Short-Form Health Survey (SF-12).

### SF-12 scale

This short version of the SF-36 tool consists of 12 items and eight scales: physical functioning (PF), role limitations due to physical problems (RP), bodily pain (BP), general health (GH), vitality (VT), social functioning (SF), role limitations due to emotional problems (RE), and perceived mental health (MH). It has 35 possible response choice indicator variables; for example, the physical functioning item has three response choice categories, 1(yes, limited a lot), 2 (yes, limited a little), 3 (no, not limited at all). The composite physical (PCS) and mental health (MCS) scores are computed using the scores of the 12 items, ranging from 0 to 100, where zero reflects the lowest health level and 100 the highest level [29]. The scoring of the SF-12 was calculated using the US norm-based scoring algorithm in SPSS software (Statistical Package for the Social Sciences) [29]. The PCS-12 and MCS-12 were computed by multiplying each indicator variable by its respective physical and mental regression weight and summing the 35 indicator variables [29]. Individual SF-12 items were recoded, summed, and transformed, with missing values imputed as recommended [29]. Subjects with missing scale scores were excluded listwise from the analysis. The Arabic version of the SF-12 was used [25], and permission to validate it was obtained from the author, Professor John E. Ware.

### Statistical analysis

Data were analyzed on SPSS software version 25. A descriptive assessment was conducted. Missing values were not replaced as they represented less than 5 % in each variable. Means and standard deviations were used for continuous variables, while counts and percentages were used for categorical variables. Comparison of means was performed using the non-parametric tests (Kruskal-Wallis and Mann-Whitney tests) since the assumption of normality was not normally distributed (p-value of the Shapiro-Wilk test < 0.05). The Spearman correlation was used for linear correlation between continuous variables. A value of *p* < 0.05 was considered significant.

Discriminant validity, convergent validity, and face validity were assessed to show how well the instrument measures the intended construct.

The validity of SF-12 was assessed by measuring the extent to which SF-12 distinguished between socio-demographic characteristics that varied by gender, age, and education level. According to previous studies, women, the elderly, and those with lower education levels have lower QOL scores.

Convergent validity was assessed using the Pearson correlation coefficient for item-scale correlations. It was expected that PF, RP, BP, and GH scores would correlate higher with PCS-12, whereas VT, SF, RE, and MH scores would correlate higher with MCS-12.

Face validity is the extent to which SF-12 is subjectively viewed as covering the concept it purports to measure. Therefore, it is expected that SF-12 would be highly associated with the presence of physical and mental illnesses among participants.

Furthermore, exploratory factor analysis (EFA) was applied to assess SF-12 structural validity using the principal component analysis with promax rotation. The Kaiser-Meyer-Olkin (KMO) measure of sampling adequacy and Bartlett’s test of sphericity were calculated to ensure the model’s adequacy. Factors with eigenvalues values greater than one were retained, and the scree plot method was used to determine the number of components to extract [30]. Only items with loading greater than 0.4 were considered [31].

Moreover, Cronbach’s alpha was recorded to evaluate reliability.

## Results

### Sociodemographic characteristics

Out of 300 adults approached, 269 (89.66 %) accepted to take part of the study. The mean age of participants was 33.38 ± 13.05 years, with 56.9 % males. Also, 174 (64.7 %) had university education level, 156 (58.2 %) were unemployed, 192 (71.4 %) were single, and 173 (65.0 %) had a low monthly income (Table 1). The PCS-12 mean score was 50.27 ± 8.94 (95 % CI: 49.18–51.36) and the MCS-12 mean score was 44.95 ± 12.17 (95 % CI: 43.47–46.43).

**Table 1:** Sociodemographic characteristics (*n* = 269)

	Frequency (percentage)
**Gender**	
Male	153 (56.9 %)
Female	116 (43.1 %)
**Marital status**	
Single	192 (71.4 %)
Married	77 (28.6 %)
**Educational level**	
Illiterate	3 (1.1 %)
Primary	13 (4.8 %)
Complementary	45 (16.7 %)
Secondary	34 (12.6 %)
University	174 (64.7 %)
**Employment status**	
Employed	112 (41.8 %)
Unemployed	156 (58.2 %)
**Monthly salary**	
Low income (< 1000$)	173 (65.0 %)
Intermediate income (1000–2000 $)	48 (18.0 %)
High income (> 2000 $)	45 (16.9 %)
	**Mean ± SD**
**Age (in years)**	33.38 ± 13.05

### Bivariate analysis

Participants with a university education level had a significantly higher mean PCS-12 score than those with a complementary education level (51.83 vs. 46.66, *p *= 0.006). Also, participants with a high income had a significantly higher MCS-12 score compared to those with low (49.86 vs. 44.02, *p* = 0.010) and intermediate (49.86 vs. 43.46, *p* = 0.010) income. Participants with physical and mental illnesses had a lower mean PCS-12 score compared to healthy individuals (46.03 vs. 52.18, *p* < 0.001, and 44.54 vs. 52.99, *p* < 0.001, respectively). Also, participants with mental illness had a lower mean MCS-12 score compared to those without mental illness (39.23 vs. 47.66, *p* < 0.001). Older age was significantly associated with lower PCS-12 (*r*=-0.249) (Table 2).

**Table 2:** Association between the sociodemographic characteristics and the SF-12 scores

	PCS – SF12		MCS – SF12	
	Mean	***p-***value	Mean	***p-***value
**Gender**				
Male	50.24 ± 9.04	0.981	44.65 ± 12.57	0.943
Female	50.30 ± 8.85		45.35 ± 11.66	
**Marital status**				
Single	50.65 ± 8.73	0.151	44.08 ± 12.25	**0.047**
Married	49.27 ± 9.44		47.18 ± 11.75	
Educational level*				
Illiterate	42.65 ± 4.48	**0.001**	39.22 ± 13.65	0.223
Primary	48.44 ± 8.05		43.99 ± 12.05	
Complementary	46.66 ± 10.87		47.56 ± 10.82	
Secondary	48.54 ± 10.19		40.41 ± 15.32	
University	51.83 ± 7.84		45.33 ± 11.66	
**Employment status**				
Employed	51.41 ± 7.74	0.307	44.74 ± 12.04	0.752
Unemployed	49.49 ± 9.66		45.04 ± 12.31	
Monthly salary*				
Low income (<1000$)	49.60 ± 9.49	0.198	44.02 ± 12.66	**0.010**
Intermediate income (1000 – 2000 $)	52.14 ± 8.39		43.46 ± 11.51	
High income (>2000 $)	50.74 ± 7.17		49.86 ± 9.80	
**Physical illness**				
Yes	46.03 ± 10.72	**<0.001**	44.68 ± 12.40	0.907
No	52.18 ± 7.27		45.07 ± 12.09	
**Mental illness**				
Yes	44.54 ± 10.06	**<0.001**	39.23 ± 12.49	**<0.001**
No	52.99 ± 6.87		47.66 ± 11.05	
	**Correlation coefficient**		**Correlation coefficient**	
Age	-0.228	**<0.001**	0.001	0.993

Bonferroni Post-hoc corrections: -Association between education level and PCS scale: illiterate vs. primary level of education *p* = 1.000, illiterate vs. complementary level of education *p *= 1.000, illiterate vs. secondary level of education *p* = 1.000, illiterate vs. university level of education *p* = 0.721, primary vs. complementary level of education *p* = 1.000, primary vs. secondary level of education *p* = 1.000, primary vs. university level of education *p* = 1.000, complementary vs. secondary level of education *p* = 1.000, complementary vs. university level of education *p* = 0.006 and secondary vs. university level of education *p* = 0.493. -Association between monthly salary and MCS scale: Low vs. intermediate income *p* = 1.000, low vs. high income *p* = 0.016, intermediate vs. high income *p* = 0.039.

### Convergent validity

All SF-12 items were significantly correlated, showing good construct validity, except for the MH subscale, where a significant correlation was found only with RE1 and VT subscales. Furthermore, the VT, SF, RE, and MH subscales were associated with MCS-12, while the PF, RP, BP, and GH subscales were related to PCS-12 (Table 3).

**Table 3:** Item- scale correlation of the SF-12 scale

	PF	RP	BP	GH	VT	SF	RE	MH	PCS	MCS
	**r, p**	**r, p**	**r, p**	**r, p**	**r, p**	**r, p**	**r, p**	**r, p**	**r, p**	**r, p**
**PF**										
** PF1**	0.848, **< 0.001**	0.364, **< 0.001**	-0.493,**<0.001**	-0.443,**<0.001**	-0.292,**<0.001**	0.335, **< 0.001**	0.169, **0.006**	0.004, 0.945	0.720,**<0.001**	0.164, **0.008**
** PF2**	0.893, **< 0.001**	0.307, **< 0.001**	-0.450,**<0.001**	-0.383, **< 0.001**	-0.221, **< 0.001**	0.320, **< 0.001**	0.207, **0.001**	-0.049, 0.425	0.639, **< 0.001**	0.185, **0.003**
**RP**										
** RP1**	0.342, **< 0.001**	0.945, **< 0.001**	-0.332,**<0.001**	-0.262,**<0.001**	-0.378, **< 0.001**	0.424, **< 0.001**	0.407, **< 0.001**	-0.116, 0.058	0.530, **< 0.001**	0.396, **< 0.001**
** RP2**	0.388, **< 0.001**	0.918, **< 0.001**	-0.346,**<0.001**	-0.305,**<0.001**	-0.366, **< 0.001**	0.431, **< 0.001**	0.423, **< 0.001**	-0.064, 0.298	0.561, **< 0.001**	0.387, **< 0.001**
**BP**										
** BP1**	-0.530, **< 0.001**	-0.350, **< 0.001**	-	0.400,**<0.001**	0.269, **< 0.001**	-0.407,**<0.001**	-0.294, **< 0.001**	0.005, 0.940	-0.679, **< 0.001**	-0.297, **< 0.001**
**GH**										
** GH1**	-0.441, **< 0.001**	-0.291, **< 0.001**	0.400,**<0.001**	-	0.307,**<0.001**	-0.392,**<0.001**	-0.196, **0.001**	-0.040, 0.519	-0.613, **< 0.001**	-0.317, **< 0.001**
**SF**										
** SF1**	0.358, **< 0.001**	0.454, **< 0.001**	-0.407,**<0.001**	-0.392,**<0.001**	-0.424, **< 0.001**	-	0.451, **< 0.001**	-0.007, 0.910	0.386, **< 0.001**	0.679, **< 0.001**
**RE**										
** RE1**	0.203, **0.001**	0.451, **< 0.001**	-0.296,**<0.001**	-0.178,**0.004**	-0.449, **< 0.001**	0.459, **< 0.001**	0.947, **< 0.001**	-0.146, **0.017**	0.061, 0.330	0.732, **< 0.001**
** RE2**	0.180, **0.003**	0.399, **< 0.001**	-0.246,**<0.001**	-0.169,**0.006**	-0.345, **< 0.001**	0.373, **< 0.001**	0.892, **< 0.001**	-0.034, 0.585	0.043, 0.485	0.623, **< 0.001**
**VT**										
**VT1**	-0.269, **< 0.001**	-0.410, **< 0.001**	0.269,**<0.001**	0.307,**<0.001**	-	-0.424,**<0.001**	-0.444, **< 0.001**	0.297, **< 0.001**	-0.303, **< 0.001**	-0.674, **< 0.001**
**MH**										
** MH1**	-0.296, **< 0.001**	-0.395, **< 0.001**	0.318,**<0.001**	0.273,**<0.001**	0.600, **< 0.001**	-0.434, **< 0.001**	-0.446, **< 0.001**	0.497, **< 0.001**	-0.184, **0.003**	-0.748, **< 0.001**
** MH2**	0.269, **< 0.001**	0.328, **< 0.001**	-0.339,**<0.001**	-0.338,**<0.001**	-0.378, **< 0.001**	0.491, **< 0.001**	0.394, **< 0.001**	0.358, **< 0.001**	0.165, **0.007**	0.726, **< 0.001**

*Spearman correlation coefficient was used. The *p*-values marked in bold are significant (less than < 0.05).

### Structural validity

All items of the SF-12 scale could be extracted from the list, and the scale converged over a two-factor solution with an eigenvalue over 1, accounting for a variance of 55.75 % (Bartlett sphericity test *P *< 0.001, KMO = 0.834). The scale components are presented in Table 4 (Factor 1: mental and Factor 2: physical). Moreover, Cronbach’s alpha values were as follows: MCS-12 (α = 0.707) and PCS-12 (α = 0.743).

**Table 4:** Factor structured of the SF-12 scale

	Factor 1	Factor 2
**Role emotional (RE**)		
Accomplished less due to emotional problems (RE)	0.920	
Not careful in work or activities due to emotional problems (RE)	0.837	
**Mental health (MH)**		
Feel calm and peaceful (MH1)	-0.729	
Feel downhearted and blue (MH2)	0.606	
**Vitality (VT)**		
Having a lot of energy (VT1)	-0.693	
**Social functioning (SF)**		
Interference of physical health or emotional problems with social activities (SF1)	0.517	
**Physical functioning (PF)**		
Limitations in moderate physical activities (PF1)		0.915
Limitations in climbing several flights of stairs (PF2)		0.823
**Role physical (RP)**		
Accomplished less due to physical health (RP1)		0.473
Limited in kind of work or activities due to physical health (RP2)		0.452
**Bodily pain (BP)**		
Pain interference with work inside or outside home (BP)		-0.678
**General health (GH)**		
Health rating in general (GH1)		-0.690
**Variance explained (%)**	42.92	12.83
**Cronbach’s alpha**	0.707	0.743

Note: Items with negative loading factors have a reversed coding.

## Discussion

This study is the first in Lebanon to validate the SF-12 Arabic version. The findings revealed that the tool is reliable and valid that can be used to determine health status. Other studies had found similar results, showing that the SF-12 scale is accurate and can be used in the general population [11, 13, 15, 18].

The mean scores of the PCS-12 (*M =* 50.27) and MCS-12 (*M = 44.95*) were comparable to the results of the Iranian and Greek studies [11, 15]. Moreover, the mental health component was lower than the physical health component in the Lebanese population, similar to previous findings [11, 15].

Regarding the sociodemographic features, our results showed that SF-12 is significantly associated with age, education level, and financial status, similar to the SF-36 scale. Older age and less education were related to worse physical health. Moreover, a higher monthly income was associated with better mental health, consistent with findings from countries such as Iran, Italy, and Greece [11, 15, 18].

The correlation of scale items with SF-12 components yielded favorable results. Expectedly, the VT, SF, RE, and MH subscales were related to MCS-12, while the PF, RP, BP, and GH were associated with the PCS-12, similar to the results found in the Iranian studies [14, 15]. Oppositely, the findings of the original paper by Ware et al. showed that vitality, general health, and social functioning were strongly correlated with both components, while PF, RP, and BP were more related to the PCS-12, and MH and RE were more associated with MCS-12 [8]. However, the Greek study conducted among 1005 participants from the general population showed that the vitality was correlated with PCS-12 and MCS-12 [11]. The controversial results found in different studies could be due to cultural differences between countries. Ware et al. reported that scales with the highest load on the physical component are more responsive to physical morbidity treatment, whereas scales with the highest mental component load often react mostly to medications and interventions that target mental health [32].

Our findings revealed that lower PCS-12 was related to physical and mental illness, and lower MCS-12 was associated with the occurrence of mental illness. Therefore, the SF-12 may provide insights into diseases, helping the practitioner make informed healthcare decisions.

Factorial analysis of the SF-12 scale with the two-factor structure yielded results identical to the original tool version [8] and those of other studies [11, 14, 15]. The internal consistency of PCS-12 and MCS-12 was favorable and comparable to other studies [11, 15, 24].

Our study could demonstrate that SF-12 is a valid and reliable tool to measure health-related quality of life in the general population.

### Limitations

Our study has several limitations. Its cross-sectional design does not allow causality to be inferred. The study results cannot be generalized to the population because of the small sample size, and the participants were mainly middle-aged, thus likely to be healthier than older adults. Information bias might have occurred since participants could not provide accurate details during the face-to-face interview. Selection bias is also possible due to the rejection rate. The test-retest reliability and convergent validity with other quality of life scales were not conducted as well. Despite these limitations, our study provides preliminary results showing that the Arabic version of the SF-12 has good psychometric properties.

## Conclusions

The Arabic version of the SF-12 is a reliable, easy-to-use, and valid tool to measure health-related quality of life in the general population. Future studies using a larger sample size and focusing on questionnaire psychometric properties are necessary to confirm our findings.

## Data Availability

Data can be made available under reasonable request form the corresponding author.

## Abbreviations

QOL: Quality of life
SF-12: 12-item short form health survey
SF-36: Short Form Health Survey 36 item
PCS-12: Physical component summary
MCS-12: Mental component summary
GH: General health perceptions
PF: Physical functioning
RP: Role limitations due to physical problems
BP: Bodily pain
VT: Vitality
SF: Social functioning
RE: Role limitations due to emotional problems
MH: Mental health
USD: United States dollar
SPSS: Statistical Package for Social Sciences
